# Adaptive transcriptomic and immune infiltrate responses in the tumor immune microenvironment following neoadjuvant chemotherapy in high grade serous ovarian cancer reveal novel prognostic associations and activation of pro-tumorigenic pathways

**DOI:** 10.3389/fimmu.2022.965331

**Published:** 2022-09-05

**Authors:** Nicole E. James, Morgan Woodman, Payton De La Cruz, Katrin Eurich, Melih Arda Ozsoy, Christoph Schorl, Linda C. Hanley, Jennifer R. Ribeiro

**Affiliations:** ^1^ Department of Obstetrics and Gynecology, Program in Women’s Oncology, Women and Infants Hospital, Providence, RI, United States; ^2^ Department of Obstetrics and Gynecology, Warren-Alpert Medical School of Brown University, Providence, RI, United States; ^3^ Pathobiology Graduate Program, Brown University, Providence, RI, United States; ^4^ Department of Biochemistry, Brown University, Providence, RI, United States; ^5^ Department of Molecular Biology, Cell Biology, and Biochemistry, Brown University, Providence, RI, United States; ^6^ Department of Pathology, Women and Infants Hospital, Providence, RI, United States

**Keywords:** tumor immune microenvironment, neoadjuvant chemotherapy, ovarian cancer, atf3, areg, NanoString, immune infiltrate, chemoresistance

## Abstract

The high rate of ovarian cancer recurrence and chemoresistance necessitates further research into how chemotherapy affects the tumor immune microenvironment (TIME). While studies have shown that immune infiltrate increases following neoadjuvant (NACT) chemotherapy, there lacks a comprehensive understanding of chemotherapy-induced effects on immunotranscriptomics and cancer-related pathways and their relationship with immune infiltrate and patient responses. In this study, we performed NanoString nCounter^®^ PanCancer IO360 analysis of 31 high grade serous ovarian cancer (HGSOC) patients with matched pre-treatment biopsy and post-NACT tumor. We observed increases in pro-tumorigenic and immunoregulatory pathways and immune infiltrate following NACT, with striking increases in a cohort of genes centered on the transcription factors *ATF3* and *EGR1*. Using quantitative PCR, we analyzed several of the top upregulated genes in HGSOC cell lines, noting that two of them, *ATF3* and *AREG*, were consistently upregulated with chemotherapy exposure and significantly increased in platinum resistant cells compared to their sensitive counterparts. Furthermore, we observed that pre-NACT immune infiltrate and pathway scores were not strikingly related to platinum free interval (PFI), but post-NACT immune infiltrate, pathway scores, and gene expression were. Finally, we found that higher levels of a cohort of proliferative and DNA damage-related genes was related to shorter PFI. This study underscores the complex alterations in the ovarian TIME following chemotherapy exposure and begins to untangle how immunologic factors are involved in mediating chemotherapy response, which will allow for the future development of novel immunologic therapies to combat chemoresistance.

## Introduction

Ovarian cancer accounts for more deaths compared to any other female reproductive cancer, with 19,880 new cases and 12,810 deaths in 2022 in the United States alone (1). The high lethality of this malignancy is attributed to the fact that patients are frequently diagnosed at an advanced stage, and that patients often experience a recurrence within 12-18 months following an initial successful frontline chemotherapeutic regimen (2). Furthermore, while many other cancer subtypes have benefited from the advances made in immunotherapy research within the last decade, the majority of ovarian cancer patients have exhibited low response rates to currently studied immunotherapies (3). Nevertheless, it has been extensively reported that ovarian tumors demonstrate anti-tumor immune responses and that cytotoxic CD8+ T cells correlate with improved survival (4–9). Therefore, it is well established that the induction of a robust anti-tumor immune response is favorable to ovarian cancer prognosis.

In addition to intratumoral T cells, recent studies have shown that immune-related gene expression profiles serve as predictive markers of response to chemotherapy and clinical outcomes in solid tumors, including ovarian cancer. Our previous work uncovered a multi-dimensional immune signature that identified patients with a long progression free survival (PFS), and specifically found that higher mRNA levels for the T cell co-receptors ICOS and LAG-3 in naïve to treatment tumors were predictive of improved patient outcomes (10). Furthermore, a plethora of studies have employed computational analysis of tumors with publicly available gene expression data to better understand the relationship between tumor immune features and prognosis (11–15). Taken together, these studies clearly demonstrate that immune-related genes and cell subsets possess prognostic capabilities in ovarian cancer.

A handful of studies have begun to characterize the effect of chemotherapy exposure on the tumor immune microenvironment (TIME) in ovarian cancer, however these investigations have centered largely upon specific immune cell population changes examined by immunohistochemistry, with many groups reporting an increase in tumor infiltrating lymphocytes (TILs) following neoadjuvant chemotherapy (NACT) (16–24). Furthermore, a recent study by Lodewijk et al. employed immunogenomic sequencing in pre- and post-NACT ovarian tumors to determine how molecular heterogeneity and homologous recombination defects relate to immune infiltration and patient outcomes (17). While these studies are an important first step in defining how the landscape of the TIME is altered following NACT in ovarian cancer, underlying mechanisms driving immune signaling within the tumor and how chemotherapy-induced immunologic changes contribute to detrimental tumor adaptations and chemoresistance and recurrence remains poorly understood. In this current study we sought to gain an in-depth understanding of tumor immune signaling, immunotranscriptomic, and immune infiltrate changes in response to NACT in high grade serous ovarian cancer (HGSOC), and determine how these responses relate to platinum free interval (PFI), with the ultimate goal of revealing novel targetable immune-based genes and signaling networks following frontline chemotherapy.

## Methods

### Patient samples

A total of 31 HGSOC patients were included in this retrospective study. Patients were selected based on pathology and clinical diagnosis of HGSOC, which by definition refers to patients with serous pathology with grade 3 disease or greater. In order to target our investigation further, we focused only on stage III and IV disease, since HGSOC is most frequently diagnosed at a late stage. Matched formalin-fixed, paraffin-embedded (FFPE) tumor tissues from treatment naive biopsies and interval debulking surgery following exposure to NACT were obtained from each patient, with associated clinical information, for a total of 62 samples. All experiments were performed in accordance with the relevant guidelines and regulations of the Women and Infants Hospital Institutional Review Board committee. All patients received frontline carboplatin and paclitaxel, although some patients received additional therapies. Treatment regimens, along with complete patient clinical information is listed in **Table 1**.

**Table 1:** Patient clinical characteristics.

	N (% of total patients)
Stage	
IIIA	1 (3%)
IIIB	3 (10%)
IIIC	23 (74%)
IV	4 (13%)
Frontline Therapy	
Carboplatin/Paclitaxel Only	14 (45%)
Additional Therapeutic Regimens*:	
GOG 3005, Veliparib or placebo	8 (26%)
GY007, Ruxolitinib	3 (10%)
Tesaro First, (TSR-042) or placebo	2 (6%)
Bevacizumab	5 (16%)
Atezolizumab	2 (6%)
Recurred	
Yes	27 (87%)
No	4 (13%)
	Median [Range]
Age at Diagnosis	63 [46-79]
Platinum free interval (PFI)	10 [1-31]

*Some patients received more than one additional therapeutic regimen.

### RNA isolation and NanoString nCounter^®^ PanCancer IO360

Pre-treatment and post-treatment cases were reviewed to select an optimal FFPE tissue block for each case. An optimal pre-treatment block contained maximum tumor cellularity (minimum of 20%) with no lymph node tissue present. An optimal post-treatment block contained maximum tumor cellularity (minimum of 20%) with no lymph node tissue present, and with evidence of a lymphocytic response. For each block, ten unstained sections of 4-5 µm thickness were cut and placed on Avantix uncharged slides. An eleventh slide was cut at 4-5 µm, stained with hematoxylin and eosin, cover-slipped and reviewed to confirm the appropriate tissue was still present. FFPE sections were scraped into tubes for RNA isolation using an RNeasy FFPE Kit (Qiagen, 73504) according to manufacturer’s instructions. RNA concentration and quality were measured by NanoDrop and 50 ng RNA was used for analysis with the nCounter PanCancer IO 360™ Panel (NanoString, XT-CSO-HIO360-12). The reporter code set and capture probe set tubes were removed from -80°C and thawed on ice prior to mixing by tapping and pulse centrifugation. 70 µl hybridization buffer was added to the tube containing the reporter probe set and mixed by tapping, followed by pulse centrifugation. 8 µl of the diluted reporter probe was added to a PCR tube. Each RNA sample was diluted in water to 25 ng/µl and 2 µl RNA was mixed with 3 µl water to 5 µl final volume for 50 ng total input per sample. 5 µl RNA sample was added to each tube containing the reporter probe set and mixed by tapping, followed by pulse centrifugation. 2 µl capture probe set was added to each tube, mixed by tapping, and the sample was collected by pulse centrifugation and then immediately placed in a pre-heated PCR machine, with the lid set to 70°C and the block set to 65°C. The hybridization was performed for 18 h at 65°C. Samples were cooled to 4°C and any condensation was collected by pulse centrifugation. Each sample was diluted with 18 µl hybridization buffer. An nCounter Sprint cartridge was calibrated to room temperature for 15 min prior to injection of 33 µl of each sample into one of the 12 injection ports of the cartridge, followed by the introduction of an air seal. The sample injection cartridge was sealed with provided tape and the reagent supply ports were unsealed prior to loading on the nCounter Sprint profiler. The analysis run was started immediately after loading. The resulting data file in RCC format was used for data analysis. RCC files were deposited in NCBI’s Gene Expression Omnibus (GEO) (25) and are accessible through GEO Series accession number GSE201600 (https://www.ncbi.nlm.nih.gov/geo/query/acc.cgi?acc=GSE201600).

### Cell culture

PEA1/PEA2 and PEO1/PEO4 cells were obtained from Millipore Sigma and cultured in RPMI 1640 supplemented with 2 mM Glutamine, 2 mM Sodium Pyruvate, and 10% Fetal Bovine Serum (FBS). OVCAR4 cells were also purchased from Millipore Sigma and cultured in RPMI 1640 supplemented with 2 mM Glutamine, 0.25 U/ml Insulin (Millipore Sigma, 407709), and 10% FBS. OVCAR8 cells were originally purchased from American Type Culture Collection (ATCC) and cultured in DMEM with 10% FBS. OV90 cells were obtained from ATCC and cultured in a 1:1 mixture of MCDB 105 medium containing a final concentration of 1.5 g/L sodium bicarbonate and Medium 199 containing a final concentration of 2.2 g/L sodium bicarbonate, supplemented with 15% FBS. All cells were cultured in 1% penicillin/streptomycin and kept in a 37 °C/5% CO_2_ humidified chamber. All cell lines were treated with 100 µM carboplatin (Santa Cruz Biotechnology, CAS 4157.5-94-4) and 10 nM paclitaxel (NIH Developmental Therapeutics Drug Cancer Panel) in combination, with control cells treated with DMSO (Sigma Aldrich, D54879) for 48 h. For chemotherapy treated cells, cells were spun down in order to retain detached and detaching cells.

### RNA isolation and quantitative PCR

RNA was isolated using Trizol extraction/LiCl high salt precipitation. Total RNA (500 ng) was reverse transcribed into cDNA using the iScript cDNA Synthesis Kit (Bio-Rad, 1708890) according the manufacturer’s protocol. All quantitative PCR was performed in triplicate by loading 1 μl of cDNA reaction, 5 μM primers, 10 μl of SYBR Green (New England Biolabs, M3003E), and 5 μl of RNAse-free water to each well. For validated BioRad primers a final 1X concentration was used with 10 μl of SYBR green (New England Biolabs, M3003E), and 8 μl of RNAse-free water added to each well. All samples were run on an ABI 7500 Fast-Real Time PCR System, and data was analyzed using the ΔΔ Ct method, with relative expression levels normalized to 18s rRNA. Validated primers were purchased from realtimeprimers.com (*ATF3, NFATC2, DUSP1*) or Bio-Rad (*AREG, SGK1*). Custom primer sequences (Invitrogen) are as follows:

18S rRNA—F-CCGCGGTTCTATTTTGTTGG

18S rRNA—R-GGCGCTCCCTCTTAATCATG

### Fluorescent immunohistochemistry

FFPE human ovarian cancer tissue slides were baked for 2 hours at 65°C. Slides were then washed in SafeClear xylene substitute, 100, 95, 70% ethanol, deoxygenated water, and FTA Hemagglutination Buffer. Antigen retrieval was performed using Antigen Retrieval Solution (1X) (Vector Laboratories, H-3300) and heated to 95°C for 20 min. Slides were then blocked with 5% horse serum in FTA Hemagglutination buffer and incubated overnight in primary antibody diluted in FTA buffer with 5% horse serum at 4°C. Anti-rabbit IgG Dylight 488 secondary antibody (Vector Laboratories, DI-488, 1:1000) was then applied to slides followed by incubation in the dark at room temperature for 1 hour. Slides were washed between each step using FTA Hemagglutination buffer and cover-slipped with DAPI containing mounting medium (Vector Laboratories, H-1200). For HGSOC cell lines, cells were cultured in chamber slides, fixed with 4% paraformaldehyde for 20 min, permeabilized in 0.1% Triton-X for 5 min, blocked in phosphate buffered saline-Tween 20 (PBS-T) with 5% horse serum for 30 min, then incubated overnight with primary antibody in the same blocking solution. Slides were washed the next day with PBS then incubated for 1 h with secondary antibody in PBS-T, washed again, and finally coverslipped with mounting media with DAPI.

Primary antibodies and dilutions used were as follows:

ATF3 (Novus Biologicals, NBP1-85816, [1:50])

AREG (Proteintech, 16036-1-AP, [1:50])

CD8 (Origene, TA802079, [1:50])

### Microscopy

Images were obtained from a Zeiss Axio Imager M1 and were acquired using diode lasers 402, 488, and 561. To obtain images for CD8+ cell counting, five randomly selected fields per sample were selected based on DAPI staining and acquired using a 40x objective. For AREG and ATF3, three randomly selected fields per sample were selected based on DAPI staining and acquired using a 20x objective. Each wavelength was acquired separately and an RGB image was created.

### Image analysis

Image processing and analysis was performed using Image J. Image analysis was performed on grayscale 8-bit images that were thresholded for specific staining, and mean and maximum intensity, along with integrated optical density was calculated. Representative images were taken using a 40x objective.

### cBioPortal

The co-expression feature was utilized from U133 microarray (n=310) data obtained from the Ovarian Cancer, TCGA Firehose Legacy cohort at cBioPortal.org (26, 27).

### Kaplan-Meier plotter

The ovarian cancer Kaplan-Meier plotter was accessed at https://kmplot.com/analysis/index.php?p=service&cancer=ovar (28) to determine the association of *ATF3* and *EGR1* with progression free survival (PFS) in stage III-IV, grade 3 serous ovarian cancer, using upper quartile as a cutoff. The probe sets were selected based on the recommended best probe set defined by Jetset algorithm.

### Analysis and statistics

Data was analyzed in nSolver Advanced Analysis Software. Raw data was uploaded to nSolver for automated normalization, background subtraction, and quality control (QC) check. All samples passed QC. Paired pre- and post-NACT samples were used to construct two groups of patient data to which an unpaired t-test was run to generate the data in the volcano plot. By performing the analysis this way the variability seen in patient data is mitigated and more robust data surrounding differentially expressed genes linked to post treatment status can be visualized. Differential expression was determined with p-values and Benjamini-Yekutieli adjusted p-values. Pathway scores are generated in nSolver as a summarization of expression level changes of biologically related groups of genes. Pathway scores are derived from the first Principle Components Analysis (PCA) scores (1^st^ eigenvectors) for each sample based on the individual gene expression levels for all the measured genes within a specific pathway. Expression levels of multiple genes comprise this first PC, with some genes having higher weight applied depending on their contribution to data variability. Cell type profiling scores are generated for immune cell types using expression levels of cell-type specific mRNAs as described in the literature (29). The cell type score itself is calculated as the mean of the log_2_ expression levels for all the probes included in the final calculation for that specific cell type. An additional level of quality control is by default performed, and markers that do not correlate with other cell type specific markers are discarded from the estimates of abundance. The software also utilizes a resampling technique to generate a significance level for confidence in the individual cell type scores, with lower p-values considered higher confidence. For our analysis, we excluded T_regs_, NK cells, and Th1 cells since there was low confidence in the accuracy of those cell type scores.

Pathway scores, cell type scores, and log transformed, normalized mRNA expression data was exported and utilized for further analyses in GraphPad Prism. Significant differences in median pathway and cell type scores between matched pre- and post-NACT samples were determined using 2-tailed Wilcoxon matched-pairs signed rank test. Significant differences in median pathway and cell type scores between ≤12 mo PFI and >12 mo PFI were determined using unpaired Mann Whitney test. The relationship between PFI and cell types, pathways, or gene expression was determined in Prism using Cox proportional hazards regression, with hazard ratios, 95% confidence intervals, and p-values reported. Pearson r-values with 2-tailed p-values were also generated in GraphPad Prism. Pathway and cell type scores do not indicate abundance of one cell type/pathway relative to another, but changes in scores between comparison groups can be relatively compared.

## Results

### Patient clinical characteristics

Thirty-one HGSOC patients were analyzed in this study, with matched tissue pre- and post-NACT. All patients received frontline therapy with carboplatin and paclitaxel in the neoadjuvant setting, and some patients received additional therapies or were enrolled in clinical trials. Twenty-seven patients had recurred at time of analysis (4 non-recurrent), and 17 patients were deceased (14 living), with at least 12 months follow-up time (**Table 1**).

### Analysis of immunoregulatory gene expression responses to NACT in HGSOC patient tissue

**Figure 1:**
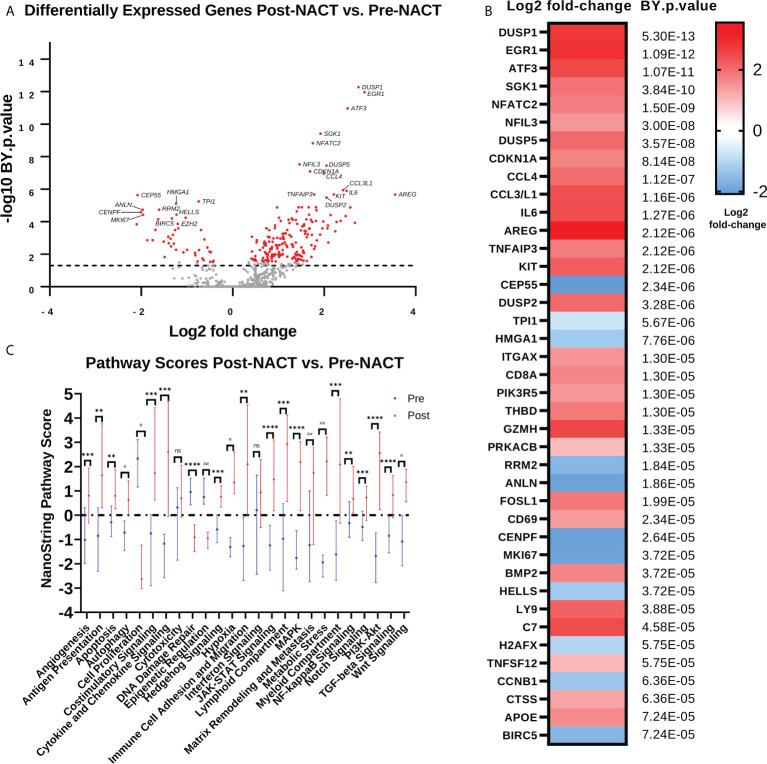
Chemotherapy promotes anti-proliferative and immunostimulatory responses but also upregulates genes that may impede the efficacy of chemotherapy. **(A)** Volcano plot illustrating differential expression of genes post-NACT relative to pre-NACT exposure in matched HGSOC tumor samples, measured by NanoString Human PanCancer IO360. Genes in red indicate Benjamini-Yekutieli (BY) adjusted p-value <0.05. **(B)** Top 40 differentially expressed genes post-NACT relative to pre-NACT, with Benjamini-Yekutieli adjusted p-value shown to the right. Heat map represents log2 fold-change. **(C)** NanoString pathway scores pre-NACT versus post-NACT, with symbols representing median scores. Significance was determined by 2-tailed Wilcoxon matched-pairs signed rank test. **p<0.005; ***p<0.0005; ****p<5e-5; #p<5e-6; ##p<5e-7; ns = not significant.

In order to determine immunologic changes resulting from NACT exposure, we performed NanoString PanCancer IO360 analysis on patient samples pre- and post-NACT. *DUSP1, EGR1, ATF3, SGK1, NFATC2, NFIL3, DUSP5, CDKN1A, CCL4*, and *CCL3*/*L1* are among the top upregulated differentially expressed genes (DEGs) post-NACT relative to pre-NACT, while *CEP55, TPI1, HMGA1, RRM2, ANLN, CENPF, MK167, HELLS, H2AFX*, and *TNFSF12* are among the top downregulated DEGs post-NACT relative to pre-NACT (**Figures 1A, B**; **Supplementary Tables 1**, **2**). Furthermore, we examined NanoString pathway changes following NACT, finding significant decreases in “Cell Proliferation”, “DNA Damage Repair”, and “Epigenetics”, and significant increases in all other pathways except for “Cytotoxicity” and “Interferon Signaling” (**Figure 1C**; **Supplementary Table 3**).

**Figure 2:**
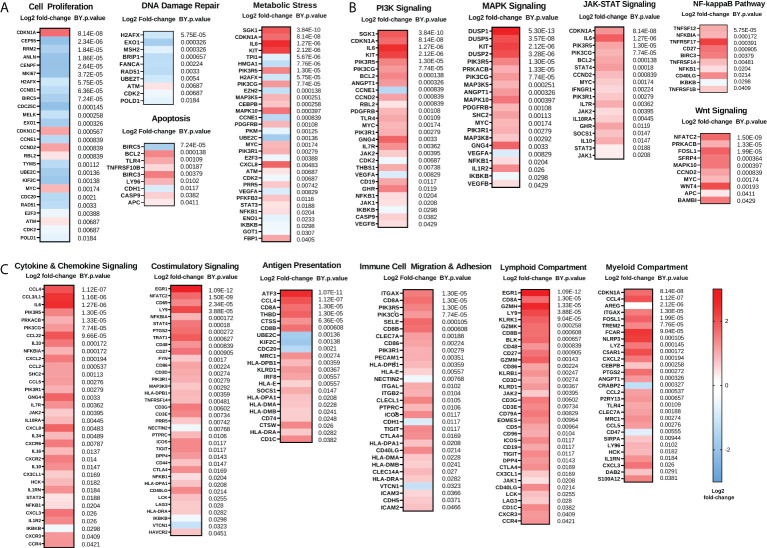
Chemotherapy downregulates proliferation and enhances immunoregulatory pathways while also upregulating pro-tumorigenic pathways. **(A)** Genes involved in proliferation and DNA damage repair pathways are generally reduced while genes involved in metabolic stress and apoptosis are generally increased following NACT. **(B)** Genes involved in pro-tumorigenic pathways such as PI3K, MAPK, JAK-STAT, NFκB, and Wnt are increased following NACT. **(C)** Genes involved in immunoregulatory pathways including cytokine and chemokine signaling, costimulatory signaling, antigen presentation, and immune cell migration and adhesion, as well as lymphoid and myeloid compartment genes, are generally increased following NACT. Heat map represents log2 fold-change of the indicated genes, with Benjamini-Yekutieli (BY) p-value listed adjacent. The heat map scale bar refers to all plots in figure.

We next examined gene changes associated with these individual pathways, which we grouped into three categories: 1) Proliferation and Stress Response (**Figure 2A**); 2) Pro-tumorigenic Signaling Pathways (**Figure 2B**); and 3) Immunoregulatory Pathways (**Figure 2C**). Pathways with fewer gene changes can be seen in **Supplementary Figure 1**. Each of these pathways displayed some overlap with other pathways. Of note, *CDKN1A* gene, which codes for the tumor suppressor protein p21, was the top upregulated gene in the “Cell Proliferation” pathway, while most other proliferation related genes were downregulated. In addition, mitogen-activated protein kinase (MAPK) signaling genes such as *DUSP1/2/5*, and *KIT* were strongly upregulated, as were Phosphoinositide 3-kinase (PI3K) signaling genes *SGK1* and *IL6*. Finally, in the Immunoregulatory Pathways category, robust increases were observed in cytokines such as *CCL4, CCL3L1*, and *IL8*, as well as transcription factors *ATF3* and *EGR1*.

These findings suggest that chemotherapy may introduce changes that have both immunostimulatory and immunosuppressive effects. Our data demonstrate that chemotherapy regulates genes and pathways important for mediating its cytotoxic effects, such as through regulation of proliferation and DNA damage, but also demonstrates upregulation of genes and pathways that may impede its efficacy or contribute to the development of chemoresistance, such as through regulation of pro-tumorigenic pathways. In addition, we observed that two of the top regulated genes are *EGR1* and *ATF3*, which are both master regulating transcription factors that may play a key role in mediating many chemotherapy-induced effects. Interestingly, while both *EGR1* and *ATF3* are well studied as stress-responsive transcription factors, *ATF3* emerged as a gene of interest since it is a master transcription factor that regulates immunity and inflammation, but little is known about its role in ovarian cancer.

### *In vitro* validation of chemotherapy-induced changes observed in human tumors

**Figure 3:**
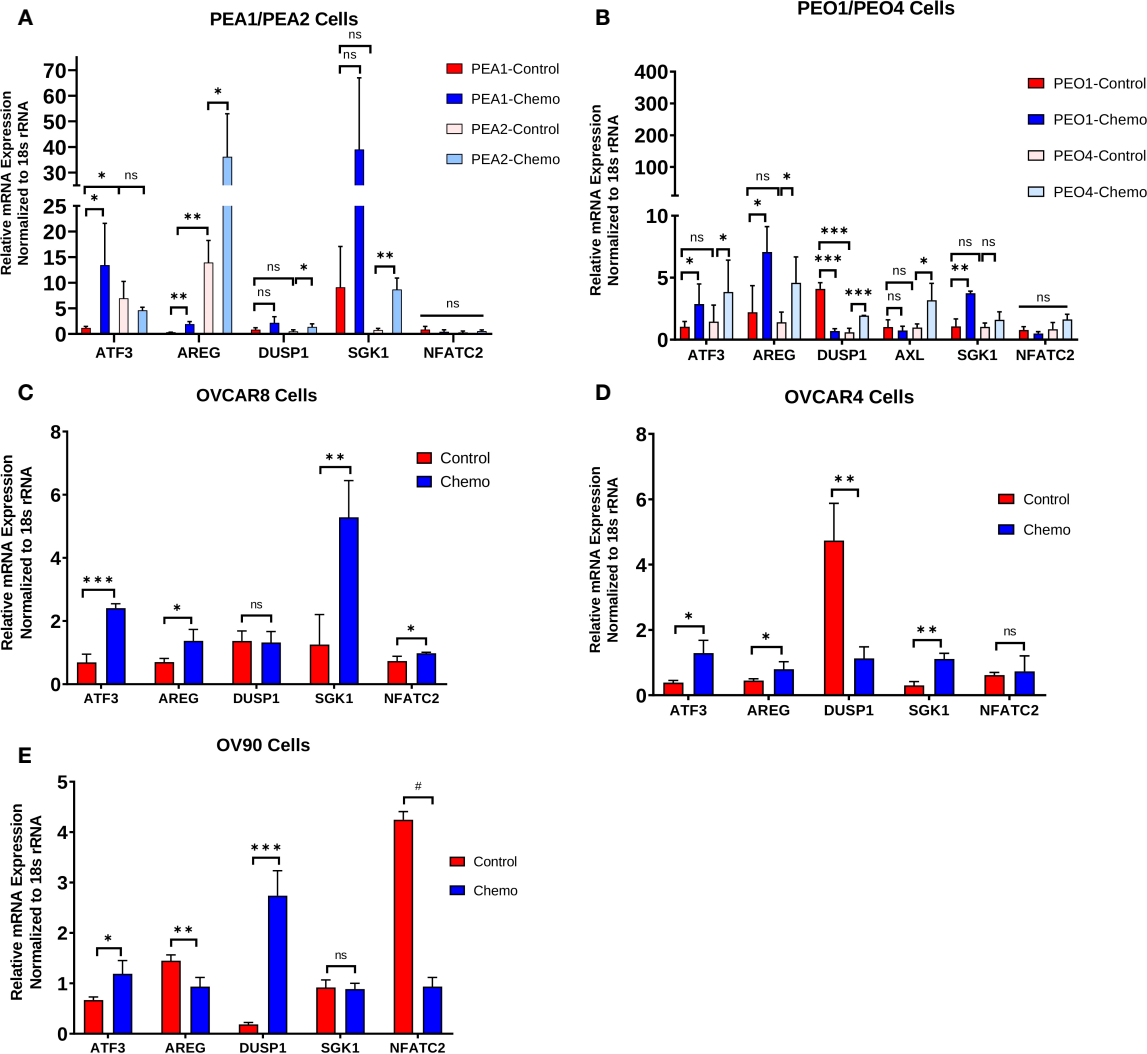
Chemotherapy-induced changes observed in human tumors are also evident in HGSOC cell lines and are associated with the chemoresistant phenotype. *ATF3, AREG, DUSP1, SGK1*, and *NFATC2* gene expression in **(A)** matched platinum-sensitive/resistant PEA1/PEA2 and cells, **(B)** matched platinum-sensitive/resistant PEO1/PEO4 cells, **(C)** OVCAR8 cells, **(D)** OVCAR4 cells, and **(E)** OV90 cells treated with 100 μM carboplatin and 10 nM paclitaxel for 48 h, measured by qPCR. Significance was determined by 1-tailed, unpaired student *t* test. *p<0.05; **p<0.005; ***p<0.0005; #p<5e-6; ns=not significant.

We examined a subset of top DEGs (*ATF3, AREG, DUSP1, SGK1*, and *NFATC2*) using HGSOC cell lines treated with carboplatin and paclitaxel chemotherapy for 48 h (**Figure 3**). Cell lines examined included the matched platinum-sensitive/resistant pairs PEA1/PEA2 and PEO1/PEO4, as well as OVCAR8, OVCAR4, and OV90 cells. Quantitative PCR revealed consistent increases in *ATF3* and *AREG* mRNA levels with chemotherapy exposure in all cell lines, except OV90 cells for *AREG*. Moreover, PEA2 chemoresistant cells displayed significantly higher *ATF3* and *AREG* levels than their chemosensitive counterpart PEA1. *DUSP1* and *SGK1* also displayed increases with chemotherapy exposure in certain cell lines, although these genes were less consistent than *ATF3* and *AREG*. Finally, *NFATC2* levels were relatively low in all cell lines except OV90, and the increase in *NFATC2* levels observed in human tumors with chemotherapy exposure was not observed in cell lines, which is consistent with its role as a T cell transcription factor.

### ATF3 and AREG protein levels following NACT exposure

**Figure 4:**
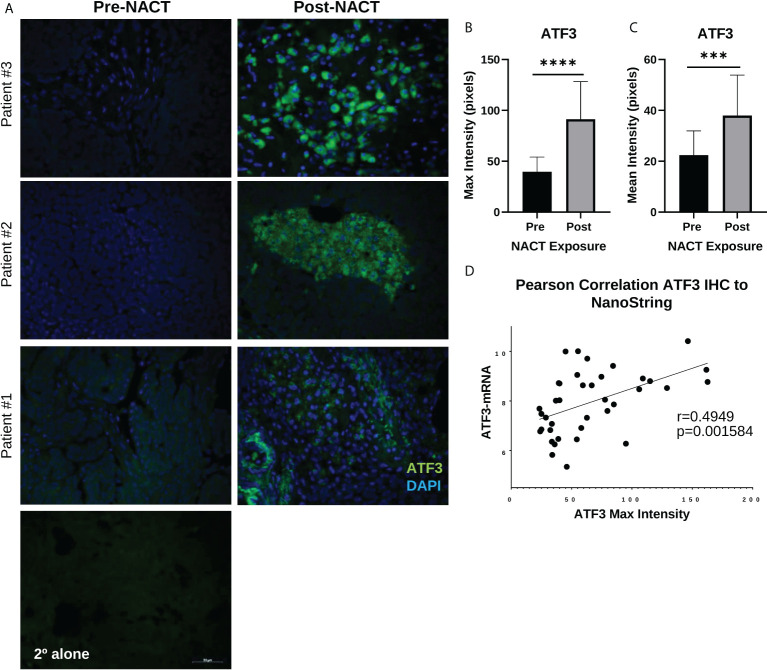
ATF3 protein displays distinct regional nuclear and cytoplasmic upregulation in human HGSOC tissue post-NACT. **(A)** Representative IHC images of ATF3 protein in three different patients pre-NACT and post-NACT. ATF3 = green; DAPI (nuclear) = blue. 2° alone = secondary antibody control. **(B)** Quantification of ATF3 maximum intensity pre-NACT and post-NACT. **(C)** Quantification of ATF3 mean intensity pre-NACT and post-NACT. Significance was calculated using 1-tailed, unpaired student *t* test (n=20 patients). ***p<0.0005; ****p<5e-5 **(D)** Pearson correlation of ATF3 maximum intensity levels with NanoString RNA values.

We next validated the increase in *ATF3* and *AREG* genes at the protein level using fluorescent immunohistochemistry (IHC) in a subset of patients from our NanoString cohort. Interestingly, we observed a robust increase in cytoplasmic and nuclear ATF3 in patients’ post-NACT, which was highly regional in occurrence (**Figure 4A**). Mean and maximum ATF3 intensity were significantly increased with NACT exposure (**Figures 4B, C**). Maximum ATF3 intensity was furthermore significantly correlated to NanoString mRNA levels for each patient, indicating the robustness of the NanoString data (r = 0.49, *p* = 0.002)(**Figure 4D**). The regional occurrence of ATF3 upregulation strongly suggests a highly heterogeneous chemotherapy response, which should be further examined using spatial profiling approaches to understand the localized responses and correlates of chemotherapy response. To further query the unexpected cytoplasmic staining observed, we performed fluorescent IHC in PEA1 and PEA2 HGSOC cell lines. In PEA1 cells, we observed the expected nuclear localization of ATF3, indicating the specificity of the antibody. However, in PEA2 cells, we observed more strong cytoplasmic expression, indicating that ATF3 can be found in the cytoplasm in addition to the nucleus, although the significance of this finding is not yet known (**Supplementary Figure 2**).

**Figure 5:**
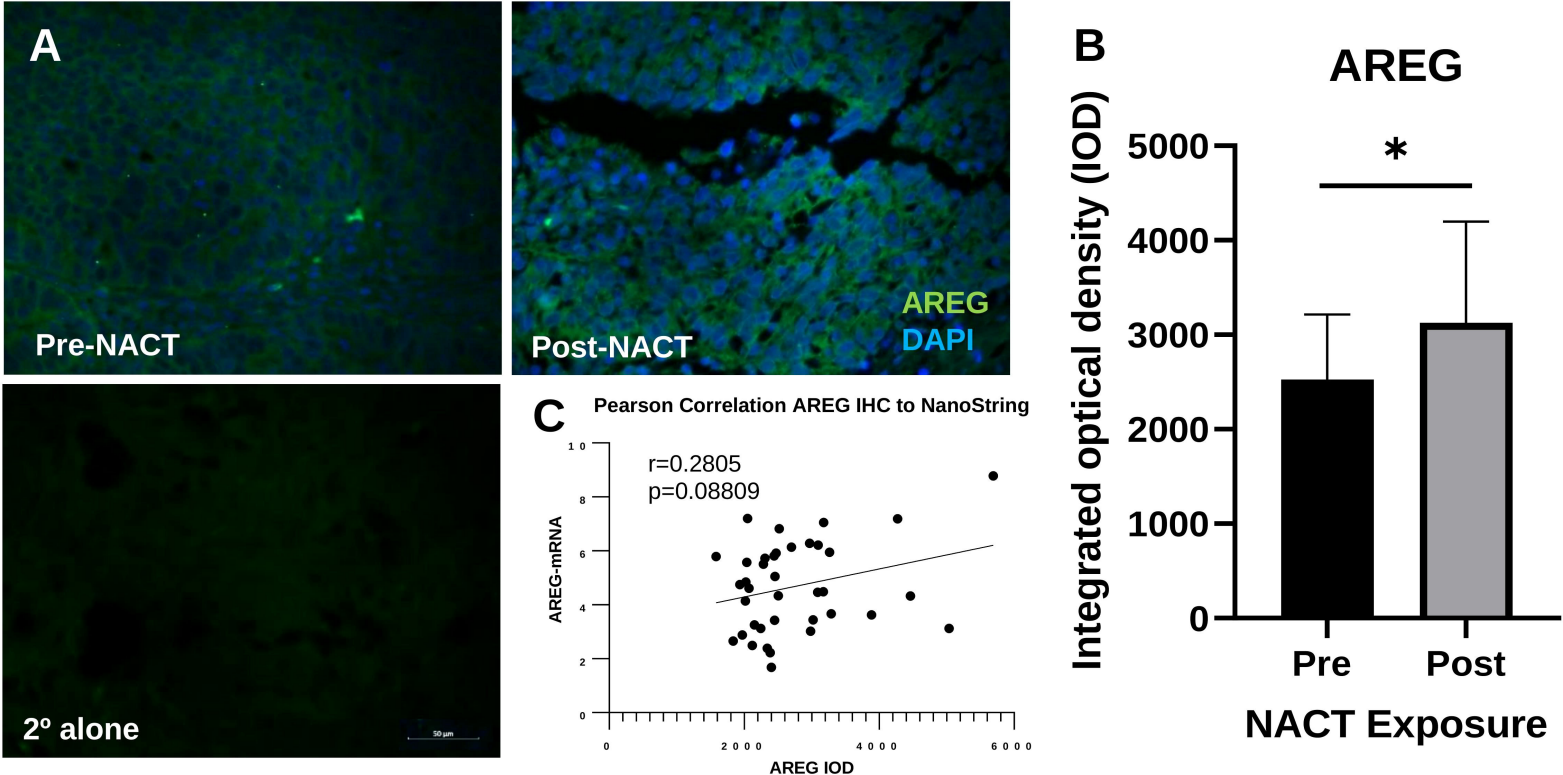
Intratumoral AREG is increased post-NACT. **(A)** Representative IHC images of AREG protein pre-NACT and post-NACT. AREG = green; DAPI (nuclear) = blue. 2° alone = secondary antibody control. **(B)** Quantification of AREG integrated optical density pre-NACT and post-NACT. Significance was calculated using 1-tailed, unpaired student *t* test (n=20 patients). *p<0.05. **(C)** Pearson correlation of ATF3 maximum intensity levels with NanoString RNA values.

We also validated the increase in *AREG* gene expression with NACT exposure at the protein level using IHC. AREG integrated optical density (IOD) was significantly increased following NACT, however this appeared less robust than for ATF3 (**Figures 5A, B**). *AREG* mRNA levels and AREG IOD were also significantly correlated, although the correlation was weaker and did not reach the level of significance, with an r-value of 0.2805 (*p* = 0.09) , which could be due to the fact that it is a secreted protein (**Figure 5C**), which could be due to the fact that it is a secreted protein.

### The cancer genome atlas and Kaplan-Meier analysis of ATF3 and EGR1

**Figure 6:**
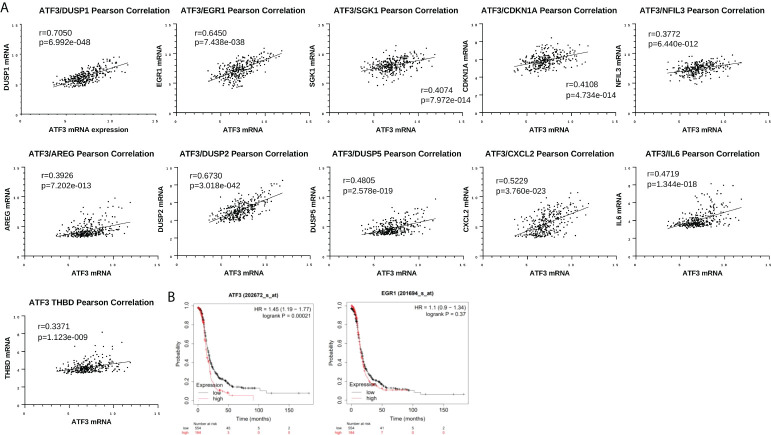
Top differentially expressed genes are associated with each other in The Cancer Genome Atlas (TCGA) but have differential relationships with patient progression free survival (PFS). **(A)** Pearson correlation between *ATF3* and *DUSP1, EGR1, SGK1, CDKN1A, NFIL3, AREG, DUSP2, DUSP5, CXCL2, IL6*, and *THBD* in cBioPortal TCGA Firehose Legacy ovarian cancer dataset (microarray U133 data, n=310). **(B)** Kaplan-Meier curves for *ATF3* and *EGR1* and PFS in Stage III-IV, grade 3 HGSOC, using top quartile cutoff (all datasets at kmplot.com).

We next went on to examine *ATF3* in the context of other top DEGs. We utilized The Cancer Genome Atlas (TCGA) Firehose Legacy ovarian cancer cohort to determine which genes ATF3 is correlated with in a large HGSOC cohort. Interestingly, many of the top ATF3 correlated genes in TCGA were among the top upregulated genes following NACT in our cohort of human HGSOC, suggesting that ATF3 is coregulated with these genes or plays a critical role in their transcriptional regulation. These genes included *DUSP1, EGR1, SGK1, CDKN1A, NFIL3*, *AREG, DUSP2, DUSP5, CXCL2, IL6*, and *THBD* (**Figure 6A**). Nonetheless, despite their strong correlations and concordant upregulation with NACT, it is important to note their potential differing functions, even among the two top upregulated transcription factors, *ATF3* and *EGR1*. As a point of comparison, we observed the relationship of gene expression levels of these two transcription factors with patient PFS using Gene Expression Omnibus (GEO) Series (GSE) and TCGA ovarian cancer data in the KMplotter (28). While *ATF3* was significantly associated with worse PFS in naïve to treatment HGSOC tumors, *EGR1* had no relationship with PFS (**Figure 6B**). While the role of these transcription factors in a stress-inducible state likely differs from their role in untreated tumors, this result demonstrates that NACT-induced *ATF3* and *EGR1* may also have very different, unique roles within the tumor microenvironment in a post-NACT setting as well.

### Analysis of immune cell infiltration responses to NACT in HGSOC patient tissue

**Figure 7:**
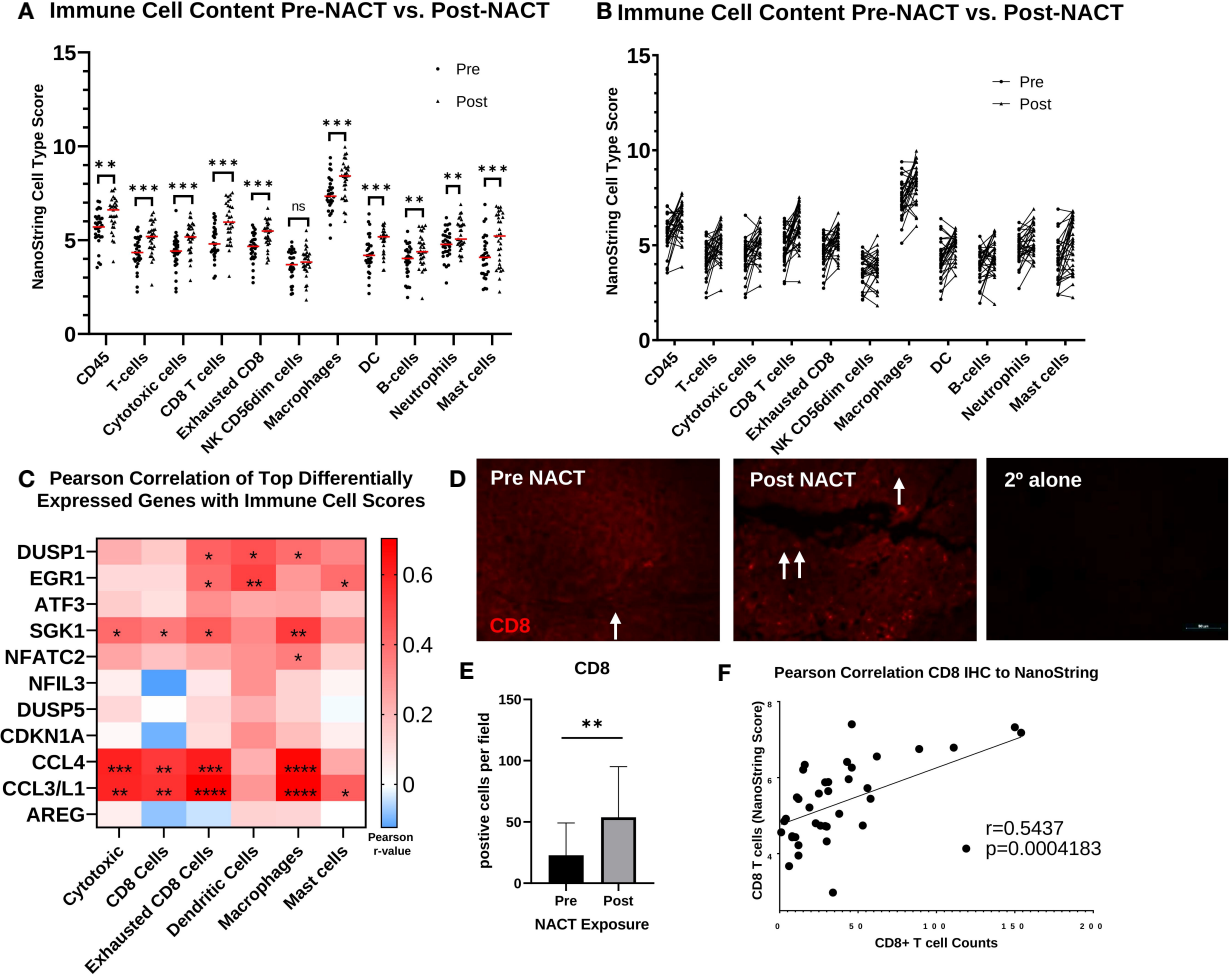
Immune cell infiltration increases post-NACT. **(A)** NanoString immune cell score changes pre- and post-NACT. Red line illustrates median. Scores do not indicate abundance of one cell type relative to another. Significance determined using 2-tailed Wilcoxon matched-pairs signed rank test. **p<0.005; ***p<0.0005; ns, not significant. DC, dendritic cells. **(B)** Individual patient changes of immune cell populations. **(C)** Pearson correlation of top differentially expressed genes post- vs. pre- NACT with immune cell population scores. *p<0.05; **p<0.005; ***p<0.0005; ****p<5e-5. **(D)** Representative image of IHC showing CD8+ T cells pre- and post-NACT. CD8 = red; DAPI (nuclear) = blue. Arrows indicate CD8+ cells. 2° alone = secondary antibody control. **(E)** Quantification of CD8+ T cell numbers pre- and post-NACT. Significance was calculated using 1-tailed, unpaired student *t* test (n=20 patients). **p<0.005. **(F)** Pearson correlation of CD8+ T cell counts with NanoString RNA values.

Next, we went on to examine immune cell infiltration changes with NACT exposure. NanoString cell type scores for all immune cell types were significantly increased following NACT, except for natural killer (NK) CD56_dim_ cells (**Figure 7A**, **Supplementary Table 4**). An examination of individual patient scores pre- and post-NACT also revealed increases for all immune cell subsets in most patients (**Figure 7B**). A correlation analysis of top DEGs with immune cell scores revealed that the cytokines *CCL4* and *CCL3L1* were strongly and significantly correlated with cytotoxic cells, CD8+ cells, exhausted CD8+ cells, macrophages, and mast cells, suggesting that these cytokines may be key mediators of immune cell infiltration post-NACT (**Figure 7C**). Finally, we validated increases in CD8+ T cells by fluorescent IHC and found significant increases in CD8+ T cell counts post-NACT (**Figures 7D, E**). We also noted a strong positive correlation between CD8+ T cell counts by IHC and NanoString CD8 cell type scores (**Figure 7F**). In sum, there is a net increase in immune cell infiltration with chemotherapy, as others have previously reported, and CCL3L1 and CCL4 could play a role in this immune cell recruitment.

### Association of immune cell infiltrate and pathway scores with platinum free interval

**Figure 8:**
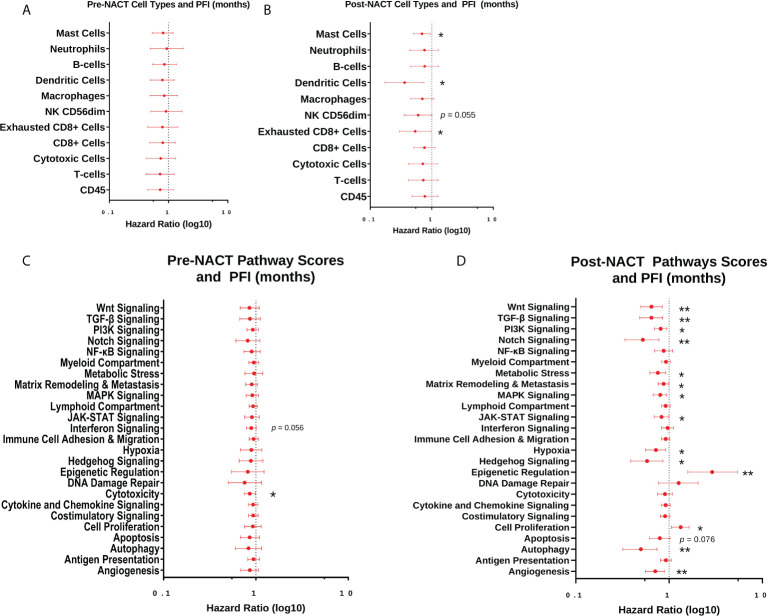
Post-NACT gene expression, pathway, and immune cell changes correlate with platinum free interval (PFI). Forest plot showing Cox proportional hazards regression of pre-NACT immune cell types **(A)**, post-NACT immune cell types **(B)**, pre-NACT pathway scores **(C)**, and post-NACT pathway scores **(D)** using PFI as the response variable. Log10 hazard ratios are indicated on the x-axis. Bars indicate 95% confidence interval. *p<0.05; **p<0.005.

Next, we performed Cox proportional hazards regression analysis of immune cell subsets with PFI, using pre- and post-NACT scores. There were no significant results with pre-NACT scores, but there was a significantly lower risk of earlier recurrence in patients with higher levels of post-NACT exhausted CD8+ cells, dendritic cells, and mast cells, while NK CD56_dim_ cells trended toward significance (**Figures 8A, B**).

When examining pre-NACT pathway scores, only the pathway “Cytotoxicity” was significantly related to lower risk of earlier recurrence. In addition, “Interferon Signaling” almost reached significance for lower risk of recurrence. On the other hand, several post-NACT pathways were significantly related to PFI. Higher scores in the categories “Angiogenesis”, “Autophagy”, Hedgehog Signaling”, “Hypoxia”, “JAK-STAT Signaling”, “MAPK Signaling”, “Matrix Remodeling and Metastasis”, “Metabolic Stress”, “Notch Signaling”, “PI3K-Akt Signaling”, “Transforming growth factor-beta (TGF-β) Signaling”, and “Wnt Signaling” were significantly associated with lower risk of recurrence. Conversely, higher scores in the pathways “Cell Proliferation” and “Epigenetic Regulation” were associated with a greater risk of earlier recurrence (**Figure 8C**).

**Figure 9:**
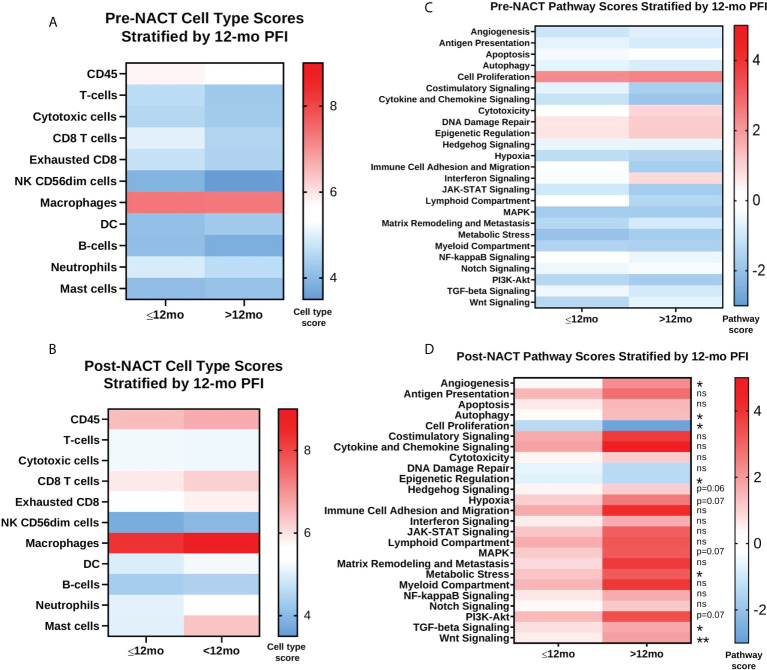
Post-NACT pathway scores are associated with platinum free interval (PFI) greater than 12 months. **(A, B)** Pre-NACT and post-NACT median cell type scores stratified by ≤12 mo PFI and >12 mo PFI. **(C, D)** Pre-NACT and post-NACT median pathway scores stratified by ≤12 mo PFI and >12 mo PFI. Significance determined by unpaired Mann-Whitney test. *p<0.05; **p<0.005; ns = not significant. Scores do not indicate abundance of one cell type/pathway relative to another.

When we stratified pre- and post-NACT samples by 12-mo PFI, we found no significant differences in median immune cell type scores (**Figures 9A, B**; **Supplementary Figures 3A, B**), despite the relationship between PFI and certain immune cell subsets observed in **Figure 8B**. Only post-NACT mast cells showed a trend toward increased levels in patients with PFI >12 mo (p = 0.07). Likewise, pre-NACT pathway scores were not significantly different between patients with ≤12 mo PFI versus >12 mo PFI (**Figure 9C**; **Supplementary Figure 3C**). However, post-NACT pathway scores were significantly different between patients with ≤12 mo and >12 mo PFI, namely for “Angiogenesis”, “Autophagy”, “Cell Proliferation”, “Epigenetic Regulation”, “Metabolic Stress”, “TGF-β Signaling”, and “Wnt Signaling”, which agrees with the results of the Cox proportional hazards regression analysis in **Figure 8D** (**Figure 9D**; **Supplementary Figure 3D**).

### Differential expression of genes post-NACT in patients with longer platinum free interval

**Figure 10:**
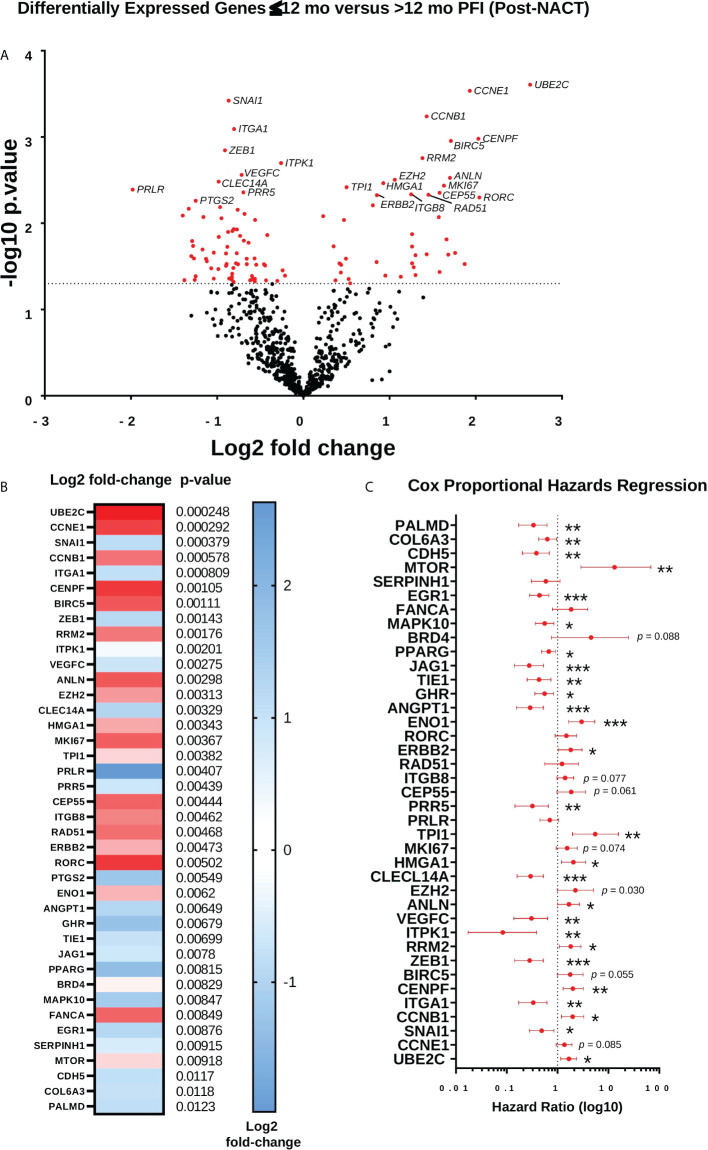
Differential expression of genes post-NACT in patients with longer platinum free interval (PFI). **(A)** Volcano plot showing differential expression of genes between patients with ≤12 mo PFI versus >12 mo PFI. **(B)** Top 40 differentially expressed genes between patients with ≤12 mo PFI versus >12 mo PFI. Heat map shows log2 fold-change, with p-value shown adjacent. No genes met Benjamini-Yekutieli adjusted p-value threshold. **(C)** Forest plot showing Cox proportional hazards regression of post-NACT gene expression and PFI. Log10 hazard ratios are indicated on the x-axis. Bars indicate 95% confidence interval. *p<0.05; **p<0.005; ***p<0.0005.

Finally, we performed differential expression analysis for post-NACT samples stratified by 12-mo PFI (**Figures 10A, B**; **Supplementary Table 5**). While this analysis did not reveal any changes that met false discovery rate correction, the genes that were differentially expressed according to p-value < 0.05 revealed a trend toward higher levels of proliferation and DNA damage repair genes in patients with PFI ≤12 mo (*UBE2C, CCNE1, CCNB1, BIRC5, CENPF, RRM2, ANLN, MK167, CEP55, RAD51*, and *FANCA*), and lower levels of expression of genes related to adhesion, angiogenesis, and epithelial-to-mesenchymal transition (*ANGPT1, ZEB1, ITGA1, SNAI1, FLT1, CDH5, PDGFRB, ICAM2, PRR5, COL17A1, VEGFC, PTGS2)* in patients with ≤12 mo PFI. Most of the genes identified through differential expression were also found to be related to PFI according to Cox proportional hazards regression (**Figure 10C**).

Together, the analysis of patient immune genes/pathways and cell types with PFI shows that patients with shorter PFI may have less robust immune infiltration of certain subsets post-NACT, although the differences are not very striking. More strikingly, post-NACT pathway scores for several typically pro-tumorigenic and immunoregulatory pathways were significantly associated with longer PFI, suggesting that these pathways may actually play a role in tumor responsiveness to chemotherapy. Finally, the trend toward higher expression of a cluster of proliferative genes, in particular *UBE2C, CCNE1*, and *CCNB1*, in patients with shorter PFI demonstrates the importance of chemotherapy-induced regulation of these genes in mediating responses.

## Discussion

In this study, we set out to comprehensively examine immunotranscriptomic and immune infiltrate changes resulting from NACT exposure in a large cohort of HGSOC patients. Our findings are mostly consistent with previous studies examining smaller cohorts of matched pre- and post-NACT tumors that reported increases in immune cell infiltration and upregulation of specific immunoreactive genes (30, 31). Our data revealed that in addition to the regulation of genes known for mediating cytotoxic effects of chemotherapy, chemotherapy exposure was also accompanied by an upregulation of genes that could impede its efficacy and/or promote chemoresistance.

Two major genes, *ATF3* and *AREG*, regulating transcription and signaling, respectively, were among the top upregulated DEGs following NACT, a finding that was validated both at the protein level and *in vitro*. Activating transcription factor 3 (*ATF3*) is induced by a variety of stress signals and regulates apoptosis as well as controls immune responses and inflammation in cancer. Our analysis derived from the ovarian TCGA cohort revealed that *ATF3* strongly correlated with many of the top upregulated DEGs from our NACT cohort, including *DUSP1, EGR1, SGK1, CDKN1A*, and *AREG*, suggesting that ATF3 may play a role in many of these genes’ transcriptional upregulation or be coordinately regulated with these genes. Intriguingly, several studies support ATF3’s direct regulation of *SGK1*, *EGR1*, *IL6*, and *CDKN1A* (32–38). *SGK1* and another top upregulated DEG, *DUSP1*, have both been implicated in chemoresistance and apoptosis inhibition in ovarian cancer (39–41), with our own research showing that a dual inhibitor of DUSP1 and DUSP6 reversed chemoresistance in ovarian cancer cells (42). These results suggest that ATF3 could regulate the balance of apoptosis and survival following NACT through transcriptional regulation of its target genes, which may include some of these top DEGs regulated by chemotherapy, although this speculation will need to be tested experimentally.

ATF3 is known to exhibit dichotomous functions both as an oncogene and tumor suppressor, depending upon cancer subtype or context of its upregulation (43). Furthermore, it has been reported that the differential usage of alternative *ATF3* promoters can lead to these dualistic roles (44). While our results from TCGA and GSE datasets show that higher *ATF3* expression in treatment naive samples is associated with worse survival, its expression in post-NACT tumors from our dataset revealed no significant association with PFI, further suggesting that ATF3’s function is highly contingent upon stress response activation. Moreover, our data uncovered specific regional upregulation of ATF3 in HGSOC patient tissue following NACT exposure, warranting further exploration into how this unique expression pattern correlates to tumor heterogeneity in chemotherapy response. Remarkably, there have been very few studies that have specifically investigated the mechanistic role of ATF3 in ovarian cancer. One bioinformatic study found that upregulation of *ATF3* was related to enhanced cell mitotic and heme-related processes (45). Interestingly, protein-protein interaction network analysis of *ATF3* DEGs identified *UBE2C* as a central hub gene (45), which we identified from our NACT dataset as a top DEG in post-NACT patient tumors stratified by ≤12 mo and > 12 mo PFI. Overall, in order to precisely understand implications of chemotherapy-induced *ATF3* expression on the ovarian TIME, future *in vitro* and *in vivo* mechanistic studies must be conducted.

In contrast to *ATF3*, the role of Amphiregulin (*AREG*), which is a low affinity ligand for epidermal growth factor receptor (EGFR), has been more established in ovarian cancer. It has been previously reported that *AREG* overexpression leads to enhanced cell proliferation, migration, invasion, cancer stemness, and drug resistance in ovarian cancer (46, 47), further supporting the idea that chemotherapy results in the upregulation of genes with pro-tumorigenic implications. Furthermore, it is well known that AREG possesses a functional role in immunity and inflammation. Specifically, AREG in T_reg_ cells acts to mitigate harmful inflammation, but simultaneously results in the promotion of tumor growth by creating an immunosuppressive TIME (48). Taken together, while AREG’s role in ovarian pathogenesis and chemoresistance has been established, AREG’s specific effects in the context of the ovarian TIME need to be elucidated, as unraveling its role in HGSOC tumor immunity could lead to novel therapeutic insights.

To further characterize immunogenomic changes resulting from NACT, pathway analysis was employed, revealing that genes involved in pro-tumorigenic PI3K, MAPK, JAK-STAT, Nuclear Factor-kappa B (NFκB), and Wnt signaling, as well as genes involved in immunostimulatory pathways such as cytokine and chemokine signaling, costimulatory signaling, antigen presentation, immune cell migration and adhesion, and lymphoid and myeloid compartment genes, were are all increased following NACT. Overall, these findings highlight the multifaceted effects that chemotherapy exerts on the ovarian TIME, as it can simultaneously produce immunostimulatory and immunosuppressive consequences. These results highlight the crucial need to study how the delicate balance of numerous pathways affect chemotherapy efficacy, and how ultimately the adaptations made by the TIME may lead to tumor recurrence. A review by Liu et al. (2020) cited studies that pointed to the potential role of the Wnt, Notch, Hedgehog, and PI3K pathways in promoting ovarian cancer stem cells, which could be key drivers of recurrence following NACT (49).

In addition to specific gene and pathway changes, we also observed significant increases in immune infiltration following NACT, which is well corroborated by previous studies (16, 17, 19, 20, 31, 50). Furthermore, we validated the finding at the protein level that CD8+ T cells were significantly increased in post-NACT tumors. Interestingly, mast cells were most strikingly increased following NACT exposure. In cancer, mast cells play a vital role in regulating the TIME through their modulation of cellular proliferation, invasiveness, metastasis, survival, and angiogenesis (51). Furthermore, a recent study in HGSOC reported that an increase in stromal tumor infiltrating mast cells (sTIMs) was correlative to an immunosuppressive subtype of HGSOC that was characterized by higher levels of T_regs_, M2 macrophages, and neutrophils and was linked to poor prognosis. Furthermore, using an organoid-patient derived model, they showed that low sTIMs were significantly associated with an increased response to anti-PD-1 treatment, indicating that mast cells could represent a novel immune target in HGSOC (52). As previously mentioned, one limitation of our study is that not all immune cell subsets could be included in the analysis, due to low confidence in accuracy of NK cell quantification and T_regs_, and the fact that the IO360 panel does not account for differences in M1/M2 macrophages. Therefore, follow-up investigations that include the analysis of these pertinent immune-cell subsets following chemotherapy exposure would be valuable.

Upon comparison of all top DEGs with immune infiltration, we discovered that *CCL4* (Chemokine (C-C motif) ligand 4) and *CCL3L1* (Chemokine (C-C motif) ligand 3-like 1) were strongly and significantly correlated with cytotoxic cells, CD8+ cells, exhausted CD8+ cells, macrophages, and mast cells. CCL4 has been reported to be involved in the metastasis, angiogenesis, and leukocyte trafficking of many tumor subtypes, including ovarian cancer (53). Corroborating our study, an investigation by Zsiros et al. similarly found a correlation between increased intratumoral *CCL4* and CD8+ T cells in ovarian cancer (54). Moreover, a study in esophageal cancer reported that CCL4 recruits cytotoxic tumor infiltrating lymphocytes (55). Finally, a study by Mlynska et al. showed that circulating levels of CCL4 could accurately identify ovarian cancer patients with shorter recurrence-free and overall survival, but found no significant association with tumor immune infiltrate (56). However, levels of CCL4 were only measured in treatment naive serum and not following chemotherapy exposure, an important distinction from our study. CCL3L1 is a key proinflammatory mediator involved in activation of leukocytes, lymphocytes, and macrophages that has been specifically implicated in glioblastoma and breast cancer tumorigenesis (57, 58) and found to be upregulated in renal cell carcinoma derived monocytes (59). Furthermore, a pan-cancer study identified *CCL3L1* as one of 20 genes indicative of T_reg_ enrichment, however this investigation only included bladder, lung, pancreatic, stomach cancer and melanoma TCGA cohorts (60). Interestingly, there has been one study in HGSOC that similarly employed a NanoString PanCancer Immune Profiling Panel to identify *CCL3L1* as being highly overexpressed in patients with chemosensitive disease (61). To the best of our knowledge, there have been no studies that have previously reported on *CCL3L1’s* potential relationship with tumor immune infiltration. Ultimately, further investigation into both CCL4 and CCL3L1’s roles in mediating tumor immune infiltration following NACT exposure is warranted, as it could potentially lead to original approaches to combat the immunosuppressive ovarian TIME.

In our analysis of how intratumoral immunotranscriptomics, pathways, and immune cell types are related to PFI in pre- and post-NACT tumors, we found significant relationships between PFI and post-NACT levels of exhausted CD8+ T cells, NK CD56dim cells, dendritic cells, and mast cells. Interestingly, we observed no significant relationships between pre-NACT levels with PFI, which contradicts previous studies that demonstrate a beneficial prognostic relationship between CD8+ T cells and survival (4–7). Other studies have also identified stronger relationships between immune infiltration and chemotherapy response than we did here. For example, Sun et al. observed enrichment of specific subsets of immune cells and greater cytolytic activity in patients with chemosensitive disease when analyzing publicly available datasets (31). Likewise, Hao et al. reported better responses in patients with higher immunoscores in TCGA and OV.AU datasets, although this was not as striking for TCGA patients (30). However, our results here are in agreement with several other studies, including one from our own lab, that demonstrated no prognostic significance of TILs alone in pre- and/or post-NACT samples (10, 18, 23, 24). Reasons for these discrepancies could be related to detection method, examination of localization and specific effector subsets of TILs in different studies, number of samples analyzed, or biological variability in patient cohorts. Moreover, despite the importance of specifically understanding the TIME in ovarian cancer, one caveat to all of these studies is that they exclude the importance of other chemotherapy-responsive genes in mediating chemotherapy resistance, which has been more thoroughly analyzed by Sun et al. in a large-scale analysis of multiple cell line and tumor databases, leading to development of a 16-gene expression signature to predict chemotherapy response (62).

When we examined pathway scores pre- and post-NACT in relationship with PFI by Cox proportional hazards regression, we again observed no relationship of pre-NACT pathways with PFI, but many post-NACT pathway scores were significantly related to PFI, and many of these were also significantly different when post-NACT samples were stratified by 12-mo PFI. Of note, while some of these pathways were logically related to chemotherapy response, such as the downregulation of “Cell Proliferation”, we also observed that activation of multiple typically “oncogenic” pathways such as Hedgehog, MAPK, Notch, PI3K, TGF-β, and Wnt signaling were associated with longer PFI. These results suggest that the role of these pathways in ovarian cancer is context dependent and may be required for mediating chemotherapy response in an acute context but could have deleterious effects that also contribute to recurrence and chemotherapy resistance. In addition to these oncogenic pathways, we also observed a relationship between “Angiogenesis” and “Matrix Remodeling and Metastasis” with PFI, suggesting that these components of the TIME are also important for mediating responses, perhaps affecting how well chemotherapy is able to perfuse the tumor.

Finally, our examination of differential gene expression between ≤12 mo and >12 mo PFI in post-NACT samples revealed no DEGs that met false discovery rate correction, which could likely be attributed to biological and treatment variability among our patient cohort and lack of power to detect such differences. Importantly, the stratification by 12-mo PFI is imperfect, as there may be patients in each group at the threshold cutoff who are more biologically similar to each other than to either group. Nonetheless, the trends observed by examining genes with p-value <0.05 were illuminating. Most prominently, the genes that emerged as more highly expressed in patients with shorter PFI were involved with cell proliferation and DNA damage *(UBE2C, CCNE1, CCNB1, BIRC5, CENPF, RRM2, ANLN, MK167, CEP55, RAD51, FANCA, MTOR*). Notably, *UBE2C* depletion has been reported to reduce platinum resistance in ovarian cancer (63). Furthermore, as mentioned above, *UBE2C* was reported as a central hub gene for *ATF3* expression in TCGA (45), suggesting that while chemotherapy-induced *ATF3* itself does not appear to be related to PFI, it may control a delicate balance of gene expression post-NACT that could negatively affect patient outcomes. Further research will be needed to identify transcriptional targets of *ATF3* following chemotherapy exposure in ovarian cancer.

On the other hand, genes that were more highly expressed in the longer PFI group were dominated by genes involved in regulating adhesion, angiogenesis and epithelial-to-mesenchymal transition (*ANGPT1, ZEB1, ITGA1, SNAI1, FLT1, CDH5, PDGFRB, ICAM2, PRR5, COL17A1, VEGFC, PTGS2*). It is unclear what the specific role of many of these genes are in ovarian cancer in the post-NACT setting, as some of them have been reported to be pro-tumorigenic in ovarian or other cancers (64–68), however these results could point to the benefit of angiogenic factors in order to promote increased drug delivery to the tumor (69). Furthermore, we noted that *EGR1* was also related to longer PFI, which indicates the importance of *EGR1* upregulation in mediating successful chemotherapy response and aligns with the role of this transcription factor as a promoter of apoptosis (70). Our own previous research found that HE4-mediated suppression of *EGR1* in ovarian cancer cells led to chemoresistance (71). Overall, these results could provide a starting point for further research into the role of many of these genes in ovarian cancer, particularly in the post-NACT setting.

In conclusion, this current study underscores the complex alterations in the ovarian TIME following chemotherapy exposure, as both immunostimulatory and pro-tumorigenic genes, pathways, and cell subsets were enriched in post-NACT patient tumors. Results derived from this investigation will require many further mechanistic studies in order to determine the role of these identified chemotherapy-induced transcriptomic and cell population changes in HGSOC, as well as to elucidate how these changes contribute to TIME adaptations that result in recurrence. Ultimately, this study begins to untangle how immunologic factors are involved in mediating chemotherapy response, which will allow for the development of novel immunologic therapies to combat HGSOC chemoresistance in the future.

## Data availability statement

The datasets presented in this study can be found in online repositories. The names of the repository/repositories and accession number(s) can be found in the article.

## Ethics statement

This retrospective study using already collected human biospecimens was reviewed and approved by the Women and Infant Hospital Institutional Review Board. No participants were contacted during the course of the study, and the ethics committee waived the requirement of written informed consent.

## Author contributions

JR and NJ developed the project conceptually, designed and conducted experiments, and performed data analysis. LH obtained all FFPE tissues and ensured tissue quality. CS performed nCounter Sprint profiling. NJ, MW, and MO performed qPCR. KE and PC performed IHC. PC assisted with statistical analysis. NJ and JR wrote this manuscript, which was reviewed and approved by all authors. All authors contributed to the article and approved the submitted version.

## Funding

Research reported in this publication was supported by a pilot award from the Women and Infants Hospital Centers of Biomedical Research Excellence (COBRE) for Reproductive Health under grant number P20GM121298; the Rhode Island Foundation; the Kaleidoscope of Hope Foundation; the Division of Gynecologic Oncology, Program in Women’s Oncology at Women & Infants Hospital; Swim Across America; the Kilguss Research Core of Women and Infants Hospital; and the Brown University Genomics Core.

## Acknowledgments

We would like to thank Dr. Christina Raker in the Division of Research at Women & Infants Hospital for statistical consultation, and Kristin O’Malley at NanoString for technical training and data analysis support.

## Conflict of interest

The authors declare that the research was conducted in the absence of any commercial or financial relationships that could be construed as a potential conflict of interest.

## Publisher’s note

All claims expressed in this article are solely those of the authors and do not necessarily represent those of their affiliated organizations, or those of the publisher, the editors and the reviewers. Any product that may be evaluated in this article, or claim that may be made by its manufacturer, is not guaranteed or endorsed by the publisher.

## Supplementary materials

Supplementary Figure 1 NACT induces gene changes in NanoString pathways. Heat map represents log2 fold-change of the indicated genes, with Benjamini-Yekutieli (BY) p-value listed adjacent. The heat map scale bar refers to all plots.

Supplementary Figure 2 ATF3 fluorescent immunohistochemistry in PEA1 and PEA2 cells. PEA1 and PEA 2 cells were stained for ATF3 (green). PEA1 cells were treated with DMSO control or 100 µM carboplatin plus 10 nM paclitaxel for 24 h prior to fixation and staining. DAPI (blue) was used as a nuclear stain. Images were taken at 20x and 40x magnification. PEA2 cells were also utilized for a secondary antibody (2°) alone control.

Supplementary Figure 3 Individual patient values for pre- and post-NACT cell types **(A, B)** and pathways **(C, D)** stratified by 12-mo PFI. Median shown with red line. Significance calculated using unpaired Mann-Whitney test. *p<0.05; **p<0.005; ns = not significant.

Supplementary Table 1 All differentially expressed genes post-NACT vs. pre-NACT.

Supplementary Table 2 Log2 transformed normalized mRNA counts, with clinical annotations.

Supplementary Table 3 Pathway scores for all samples, with clinical annotations.

Supplementary Table 4 NanoString raw cell type scores, with clinical annotations.

Supplementary Table 5 All post-NACT differentially expressed genes between patients with ≤12 mo PFI versus those with >12 mo PFI.
