# Supplementary figures and images for: Adaptive transcriptomic and immune infiltrate responses in the tumor immune microenvironment following neoadjuvant chemotherapy in high grade serous ovarian cancer reveal novel prognostic associations and activation of pro-tumorigenic pathways
Source: Front Immunol. 2022 Sep 5;13:965331. doi: 10.3389/fimmu.2022.965331 (PMC9483165; doi:10.3389/fimmu.2022.965331)

Figure S1

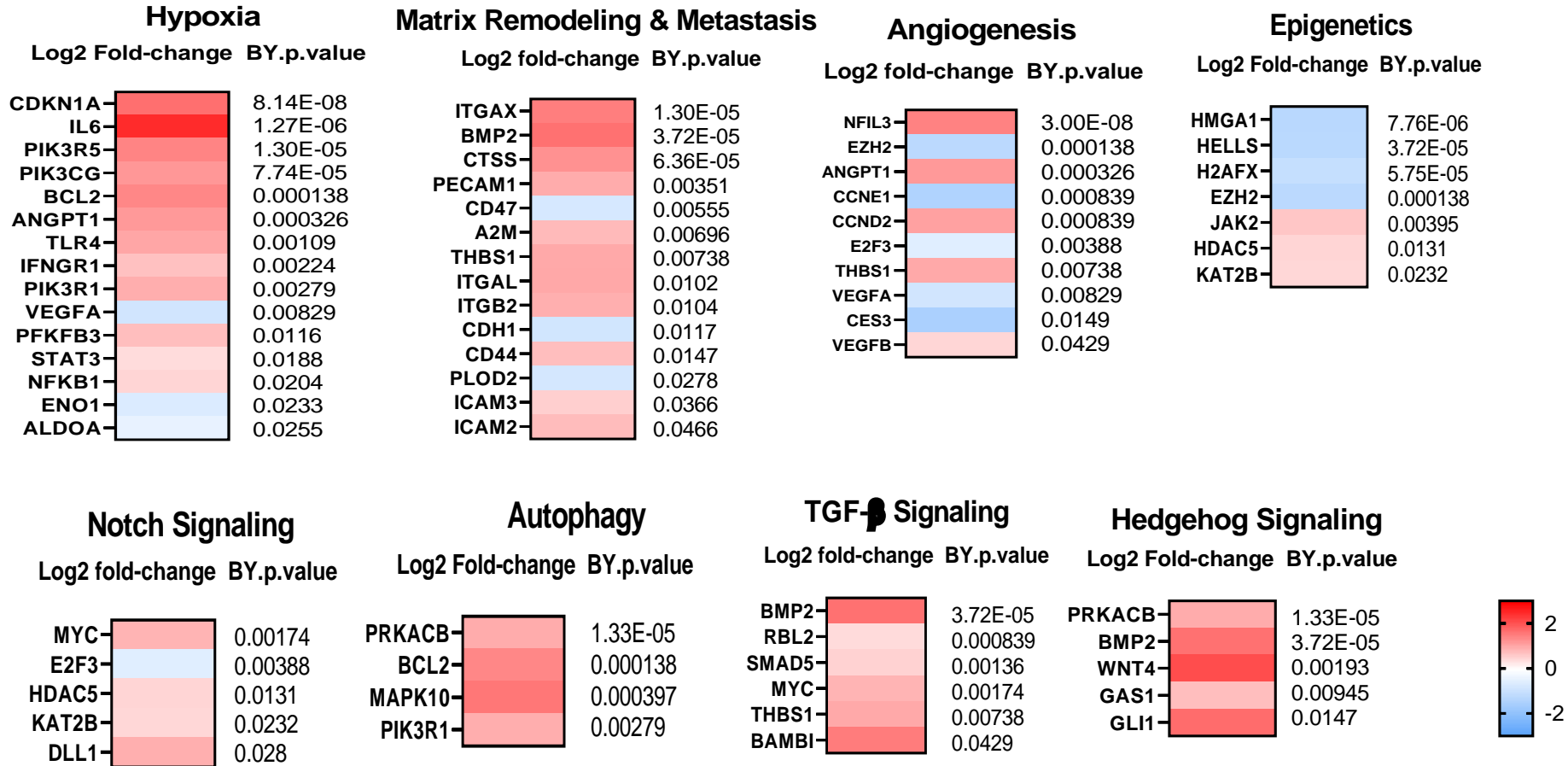

Figure S2

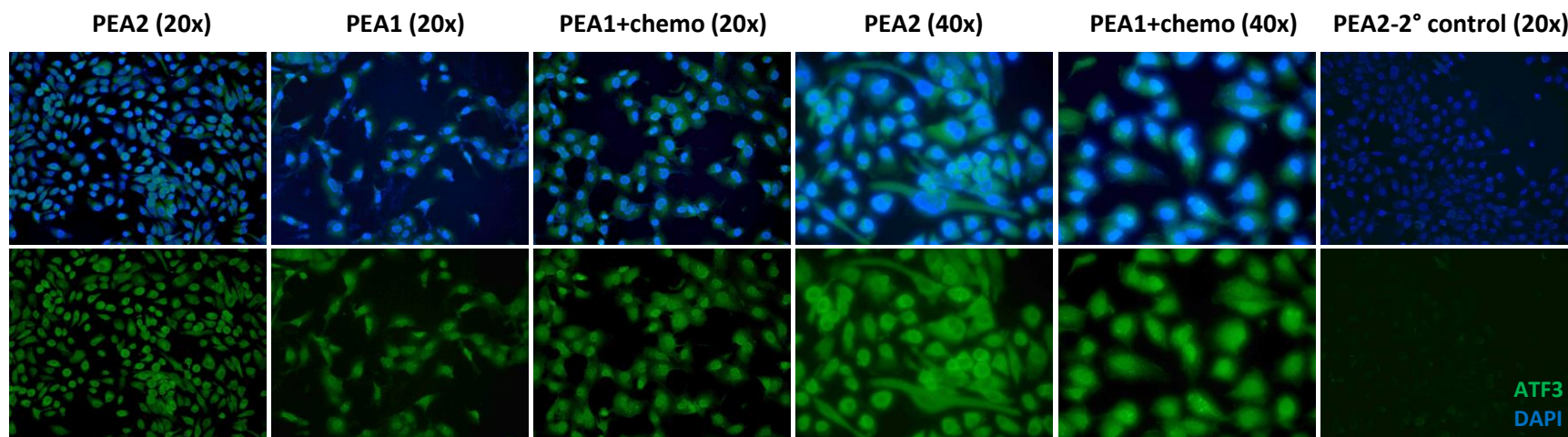

### Figure S3

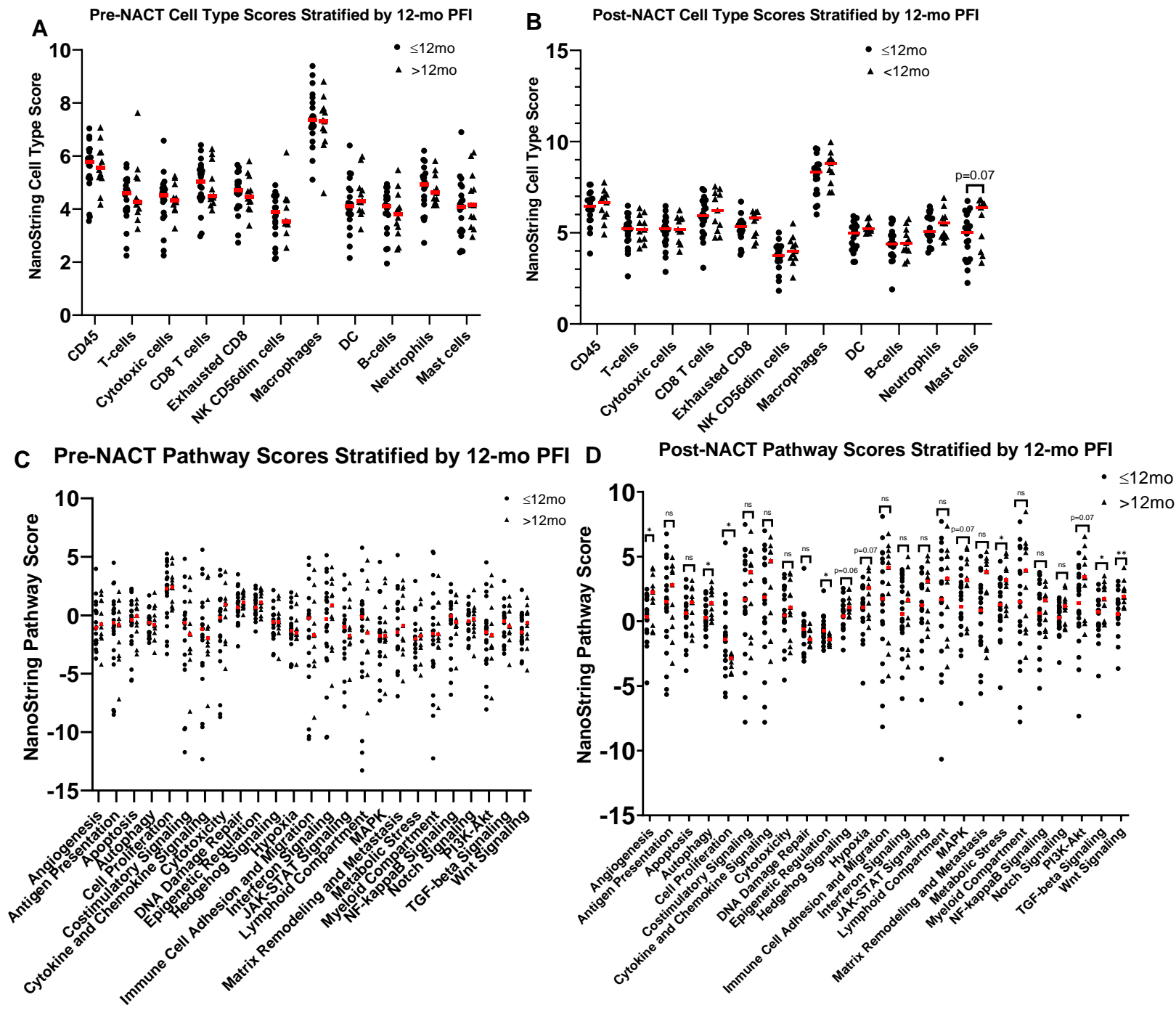

Supplement: Supplementary Figure 1 — NACT induces gene changes in NanoString pathways. Heat map represents log2 fold-change of the indicated genes, with Benjamini-Yekutieli (BY) p-value listed adjacent. The heat map scale bar refers to all plots. [file DataSheet_1.pdf]
